# The Regulation of O_2_ Spin State and Direct Oxidation of CO at Room Temperature Using Triboelectric Plasma by Harvesting Mechanical Energy

**DOI:** 10.3390/nano11123408

**Published:** 2021-12-16

**Authors:** Xue Shi, Sumin Li, Bao Zhang, Jiao Wang, Xiaochen Xiang, Yifei Zhu, Ke Zhao, Wanyu Shang, Guangqin Gu, Junmeng Guo, Peng Cui, Gang Cheng, Zuliang Du

**Affiliations:** 1 Key Lab for Special Functional Materials, Ministry of Education, National & Local Joint Engineering Research Center for High-Efficiency Display and Lighting Technology, School of Materials Science and Engineering, Collaborative Innovation Center of Nano Functional Materials and Applications, Henan University, Kaifeng 475004, China; shixuehenandaxue@126.com (X.S.); amernsuli1981@163.com (S.L.); zhangbao@henu.edu.cn (B.Z.); 13203449093@163.com (J.W.); xiang17737488621@163.com (X.X.); kezhao_5@163.com (K.Z.); shangwanyu0525@163.com (W.S.); guguangqin@vip.henu.edu.cn (G.G.); junmengguo@163.com (J.G.); cuipeng@henu.edu.cn (P.C.); zld@henu.edu.cn (Z.D.); 2 Institute of Aero-Engine, School of Mechanical Engineering, Xi’an Jiaotong University, Xi’an 710049, China; yifei.zhu.plasma@gmail.com

**Keywords:** triboelectric nanogenerator, mechanical energy, dioxygen activation, triboelectric corona plasma, O_2_^−^ reactive species, spin conversion

## Abstract

Oxidation reactions play a critical role in processes involving energy utilization, chemical conversion, and pollutant elimination. However, due to its spin-forbidden nature, the reaction of molecular dioxygen (O_2_) with a substrate is difficult under mild conditions. Herein, we describe a system that activates O_2_ via the direct modulation of its spin state by mechanical energy-induced triboelectric corona plasma, enabling the CO oxidation reaction under normal temperature and pressure. Under optimized reaction conditions, the activity was 7.2 μmol h^−1^, and the energy consumption per mole CO was 4.2 MJ. The results of kinetic isotope effect, colorimetry, and density functional theory calculation studies demonstrated that electrons generated in the triboelectric plasma were directly injected into the antibonding orbital of O_2_ to form highly reactive negative ions O_2_^−^, which effectively promoted the rate-limiting step of O_2_ dissociation. The barrier of the reaction of O_2_^−^ ions and CO molecular was 3.4 eV lower than that of O_2_ and CO molecular. This work provides an effective strategy for using renewable and green mechanical energy to realize spin-forbidden reactions of small molecules.

## 1. Introduction

**Figure 1:**
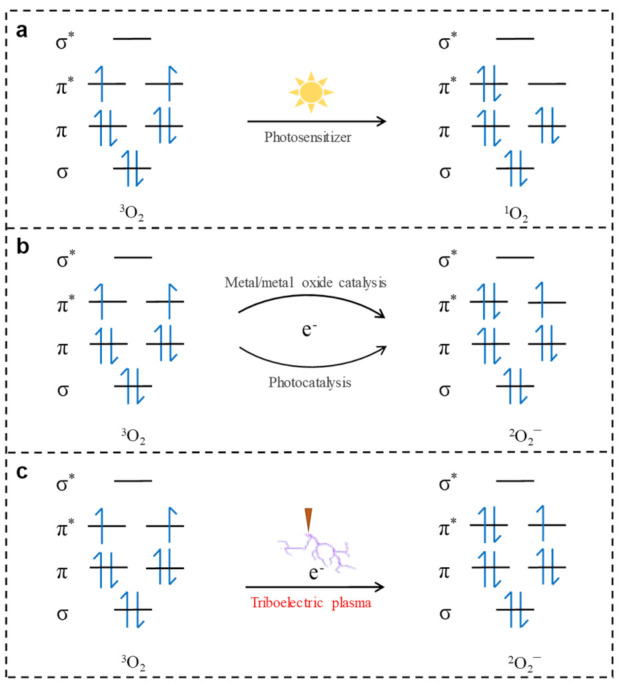
Spins conversion of molecular dioxygen using different methods. (**a**) Transformation of ^3^O_2_ to ^1^O_2_ under light irradiation with a photosensitizer. (**b**) Transformation of ^3^O_2_ to ^2^O_2_^–^ under metal/metal oxide catalysis and photocatalysis. (**c**) Transformation of ^3^O_2_ to ^2^O_2_^–^ using triboelectric plasma.

Molecular dioxygen (^3^O_2_) is the most green, pollution-free, and cheap terminal oxidant [1,2,3,4,5,6,7,8]. Unfortunately, ground state ^3^O_2_ is usually chemically inert under normal temperature and pressure conditions, and the oxidation of compounds by ^3^O_2_ is hindered by its spin-forbidden nature [1]. To enable the activation of ^3^O_2_ at room temperature, considerable research efforts have been directed toward the regulation of its spin state. As a result, a number of approaches have been developed, such as the conversion of the triplet state of dioxygen (^3^O_2_) into singlet dioxygen (^1^O_2_) via external stimulation, e.g., by light irradiation [2,3,9,10]. This can be achieved under the assistance of a photosensitizer, as shown in Figure 1a. Under light illumination, the photosensitizer absorbs energy and is excited to an excited single state, which then undergoes intersystem crossing to produce an excited triplet state. The latter transfers its energy to ^3^O_2_, which is converted to ^1^O_2_, while the excited photosensitizer returns to its ground state. The two spin-paired valence electrons of ^1^O_2_ are in one π orbital, while the second π* orbital is empty, thereby lifting the spin blockade of ^3^O_2_. Another method for the activation of ^3^O_2_ consists of its one-electron reduction to superoxide ^2^O_2_^−^ (Figure 1b) [2,4,5,6,7,8], in which the electron is transferred to the ^3^O_2_ molecule via metal/metal oxide catalysis or photocatalysis. The lowest unoccupied molecular orbital (LUMO) π* of ^2^O_2_^−^ has a single electron, which endows the molecule with free radical reactivity, easily reacting with a substrate. Nevertheless, both ^3^O_2_ activation systems require the use of metals or metal oxides as catalysts or additives [1,2,3,4,5,11,12,13,14,15], with the concomitant heavy metal pollution and economic infeasibility of large-scale production. Thus, the development of an efficient system for the activation of ^3^O_2_ for aerobic oxidation via the direct regulation of its spin state is still a huge challenge.

In this context, mechanical energy can be envisaged as an attractive source of activation energy because it is a ubiquitous, earth-abundant, and renewable energy that exists in various forms including wind energy, raindrop energy, tidal energy, and hydropower [16,17,18,19,20,21,22,23]. Compared with other renewable energy sources such as solar energy, mechanical energy offers the advantage of abundant reserves that are not affected by geographical location and weather [24,25]. In recent years, triboelectric nanogenerators (TENGs) have emerged as novel mechanical energy collection devices with broad application prospects in energy collection [26], gas sensing [27], and other fields [25,28,29]. A TENG is characterized by an advantageous high output voltage, which can directly generate a gas discharge forming triboelectric plasma [30,31]. During such a process, a large number of electrons generated through the avalanche effect can be captured by electronegative ^3^O_2_ molecules; hence, electrons are injected into the LUMO energy level of ^3^O_2_, enabling its conversion into ^2^O_2_^−^. Therefore, numerous electrons generated in the mechanical energy-induced triboelectric plasma are directly transferred into the LUMO energy level of ^3^O_2_, which is expected to overcome the spin-forbidden nature of its activation at room temperature (Figure 1c).

Herein, we report the use of triboelectric corona plasma for the direct modulation of the spin state of ^3^O_2_, which enabled its activation for the direct carbon monoxide (CO) oxidation at room temperature and pressure. We selected the CO oxidation reaction as a model reaction to study the effect of ^3^O_2_ spin change on its reactivity because it is a common model reaction of great significance in the fields of automobile exhaust treatment, fuel cells, and environmental protection. We used the high output voltage of a TENG to trigger the generation of triboelectric plasma from a gas and investigated the influence of the rotational speed of the TENG, the needle-plate distance, and the corona polarity on the triboelectric plasma. The optimal activity was 7.2 μmol h^−1^, and the minimum energy required to convert 1 mol CO was 4.2 MJ. The results of colorimetry, kinetic isotope effect (KIE), and density functional theory (DFT) studies showed that high-energy electrons generated in the triboelectric plasma were directly injected into the π* antibonding orbital of ^3^O_2_ to realize the activation of the O–O bonds at low temperature. This work provides a novel pathway for using mechanical energy to overcome the spin-forbidden transition of small molecules.

## 2. Materials and Methods

### 2.1. Carbon Monoxide (CO) Oxidation Triggered by Mechanical Energy-Induced Triboelectric Plasma

The system for the triboelectric plasma-triggered CO oxidation consisted of three parts: a TENG, a plasma oxidation reaction device, and an electrical test system. The freestanding rotating TENG with a diameter of 25 cm was composed of 10 μm of polytetrafluoroethylene (PTFE) film and a 30 μm Cu film as triboelectric layers and an electrode. The Cu and PTFE layers were adhered to polymethylmethacrylate. Two Cu films were connected to form the TENG electrodes through a rectifier bridge of the external circuit using Cu wires. When the triboelectric layers of the TENG were in contact with each other, opposite triboelectric charges were generated. When the PTFE triboelectric layer was moving, the two Cu electrodes generated a potential difference. The electrode of the triboelectric plasma was composed of a W needle and a Pt electrode plate. The radius of curvature of the W needle was 5 μm. The two discharge electrodes were located in a 500 mL reaction bottle, and the distance between the W needle and the Pt electrode plate was precisely adjusted using a three-dimensional moving platform.

The triboelectric plasma-triggered CO oxidation reaction was conducted in a self-made glass reactor (500 mL) under normal temperature and pressure. Using the TENG as the driving force, the output signal was recorded in an ammeter (Keithley 6514) and a voltmeter (Tektronix). The W needle and the Pt electrode plate (Tianjin Aida Hengsheng Technology Development Co., Ltd.,Tianjin, China, purity 99.99%) were fixed in the glass reactor, which then was purged with a mixed gas of CO/O_2_/He (1:20:79) (Henan Yuan Zheng Technology Development Co., Ltd., Kaifeng, China) for 10 min, and the air was replaced with the mixed gas three times. During the reaction, a carbon dioxide (CO_2_) detector was used to determine the CO_2_ concentration generated (Haipa air detector, accuracy 1 ppm), the reaction time was set to 1 h, the needle–plate distance was 3 mm, and the rotational speed of the TENG was 400 rpm. The effects of rotational speed of the TENG, needle-plate distance, the corona polarities, and working electrodes on the CO oxidation reaction activity were investigated.

### 2.2. Detection of ^2^O_2_^−^ Radicals

Nitro blue tetrazolium (NBT; Shandong West Asia Chemical Co., Ltd., Dongying, China) was reduced by O_2_^−^ radicals to produce a blue product, which was insoluble in water [32]. The change in the absorbance of the solution was measured using ultraviolet-visible spectroscopy to detect the existence of reactive O_2_^−^ radicals. The NBT aqueous solution had a maximum absorption peak at a wavelength of 260 nm. In a 3 mL glass reaction device, 2 mL of a 0.05 mmol L^−1^ NBT solution (V_water_:V_propanol_ = 98:2) was added. A Cu mesh electrode was placed above the solution surface to avoid the quenching of highly energetic radicals, purged with CO/O_2_/He mixed gas, and then discharged for 30 min.

### 2.3. Isotope Labeling Experiment

The triboelectric plasma-triggered CO oxidation reaction was performed using ^13^CO/^18^O_2_/Ar and ^12^CO/^16^O_2_/Ar for 10 h. The composition and concentration of the isotopic products were determined by Wuhan Newrad Special Gas Co., Ltd., Wuhan, China.

### 2.4. Isotope Kinetics Effect (KIE) Experiment

The KIE experiments were conducted using CO/^16^O_2_/He and CO/^18^O_2_/He as the substrates under the conditions of the triboelectric plasma-triggered CO oxidation reaction for 1 h at room temperature and pressure. The KIE was calculated on the basis of the reaction rates obtained using ^16^O_2_ and ^18^O_2_ as reactants.

### 2.5. Density Functional Theory (DFT) Calculations

DFT calculations were performed using the Vienna Ab-initio Simulation Package (VASP) [33,34], taking advantage of the Projected Augmented Wave (PAW) [35] method. The Perdew–Burke–Ernzerhof functional was used to describe the exchange and correlation effects [36]. For all the geometry optimizations, the cutoff energy was set to 450 eV. Spin-polarization calculations were included in all cases. Singlet and triplet oxygen were modeled by controlling the numerical difference between spin-up electrons and spin-down electrons. The simulation box was constructed as a 21 Å × 22 Å × 20 Å box. The DFT calculation process was provided by the Cailiaoren APP.

### 2.6. Triboelectric Plasma Simulation

Triboelectric plasma simulation was performed using 2D PASSKEy code (PArallel Streamer Solver with KinEtics), which was used in modelling nanosecond surface discharges and proved by discharge morphology, propagation velocity, voltage–current curves of triboelectric plasma, and a point-to-plane configuration generated from the experiments [37,38,39,40]. Moreover, 0D model global plasma chemistry code ZDPlasKin was also utilized in a house parameter reconstruction module [41].

## 3. Results and Discussion

### 3.1. CO Oxidation System Driven by Mechanical Energy-Induced Triboelectric Plasma

**Figure 2:**
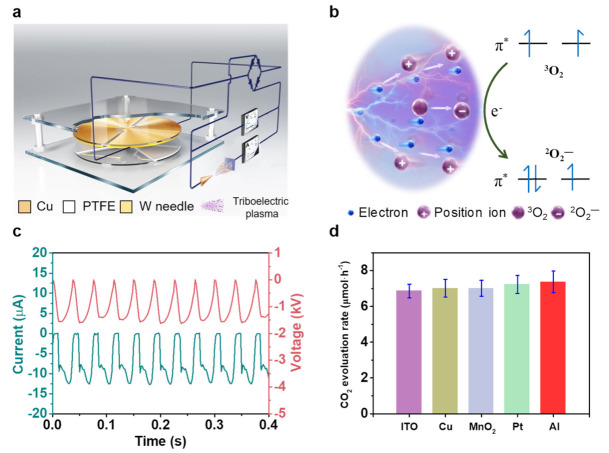
Schematic diagrams of the triboelectric plasma system. (**a**) Scheme of the triboelectric plasma oxidation system directly driven by a triboelectric nanogenerator (TENG). (**b**) ^3^O_2_ spin conversion during the triboelectric plasma-induced process. (**c**) Electrical curves of the CO oxidation triggered by triboelectric plasma with a needle–plate distance of 3 mm using Pt as the electrode plate. (**d**) Activity for the triboelectric plasma-triggered CO oxidation with different electrode plate materials. Reaction conditions: negative corona, 3 mm needle–plate distance, 400 rpm rotation speed, CO/O_2_/He (1:20:79), 1 h discharge time, room temperature, and atmospheric pressure.

As shown in Figure 2a and Figure S1, the designed system for CO oxidation driven by mechanical energy-induced triboelectric plasma consisted of three parts: a TENG, a plasma oxidation reaction device, and an electrical test system. The TENG, which was composed of a Cu film and a polytetrafluoroethylene (PTFE) film friction layer, was used as the driving force of the reaction. The plasma oxidation reaction device comprised a needle-plate electrode and a CO mixture gas (CO/O_2_/He = 1:20:79). The electrical test system consisted of a voltmeter and an ammeter. When the PTFE film was placed in contact with the Cu film, the surfaces of the two materials generated an equal amount of negative and positive triboelectric charge. By periodically rotating the PTFE film, a periodic potential difference was generated between the two Cu electrodes of the TENG, and such a potential difference was transformed into a direct current output signal by passing through a rectifier bridge. An external ammeter and voltmeter measured the electrical characteristics of the triboelectric plasma. The output voltage of the TENG (about 7.1 kV; Figure S2) was much greater than the threshold voltage of the CO mixture gas, which facilitated the discharge of the latter. For needle–plate distances (***d***) of 3–11 mm, the TENG triggered the CO mixture gas discharge and generated the triboelectric corona plasma. When the ***d*** value was less than 3 mm, the corona discharge signal was transformed into multiple pulse discharge signals. The ^3^O_2_ spin conversion during the triboelectric plasma process is depicted in Figure 2b. Free electrons near the tip of the W needle were accelerated in the electric field to form high-energy electrons [42,43,44,45], which then collided with the gas in the gap, generating the triboelectric plasma composed of a large number of electrons and positive ions via the avalanche effect. In the plasma region, some high-energy electrons were captured by the electronegative ^3^O_2_ gas, and the ^3^O_2_ molecules were transformed into reactive superoxide ions ^2^O_2_^−^, which could overcome the spin prohibition to realize the CO oxidation reaction at room temperature.

Figure 2c displays the electrical curve obtained for the CO oxidation system using triboelectric plasma generated by a TENG having a ***d*** of 3 mm. A pulse voltage peak of about 1.6 kV accompanied by a discharge current peak of 12.5 μA was generated in half a cycle. The time of the current pulse peak was 28 ms, which corresponds to the generation of triboelectric corona plasma. Figure 2d summarizes the CO oxidation activity of different electrode materials using the triboelectric plasma described in Figure 2c under normal temperature and pressure conditions. Almost all of the electrode materials showed a high CO oxidation activity of 6.9–7.4 μmol h^−1^. An ^18^O isotope labeling experiment confirmed that ^3^O_2_ participated in the CO oxidation reaction (Figure S3). These results indicate that the CO oxidation was mainly controlled by the triboelectric plasma, whereas the electrode materials had a minor effect on the CO oxidation activity. Thus, we selected Pt as the electrode for the following experiments due to its stable performance.

### 3.2. Influence of Different Parameters on the CO Oxidation Activity

The rotational speed of the TENG, the needle–plate distance, and the corona polarity affect the state of the triboelectric corona plasma and hence the activity of the oxidation reaction between ^2^O_2_^−^ and CO. Taking negative corona as an example, we first investigated the effect of the rotational speed of the TENG on the CO oxidation activity, as shown in Figure S4. Upon increasing the rotational speed, the discharge voltage was almost maintained at −1.6 kV, whereas the absolute value of the discharge current, the average power, and the CO oxidation activity increased from 2.9 μA, 1.0 mW, and 1.4 μmol h^−1^ to 14.7 μA, 9.2 mW, and 8.9 μmol h^−1^, respectively. The energy consumption (EC) of the conversion of 1 mol CO remained virtually unchanged at 4.2 MJ mol^−1^, indicating that the rotational speed has a weak effect on the EC of the CO oxidation reaction. Therefore, we selected 400 rpm as the rotational speed of the TENG for the subsequent experiments.

Figure S5 shows the relationship between ***d*** and the CO oxidation activity. As the ***d*** value increased from 3 to 11 mm, the absolute value of the discharge voltage increased from 1.6 to 3.9 kV, and the absolute value of the discharge current decreased from 12.5 to 5.7 μA. The average power decreased from 8.9 to 2.5 mW, the activity dropped from 7.2 to 0.2 μmol h^−1^, and the corresponding EC increased significantly from 4.2 to 45.4 MJ mol^−1^. According to these results, the optimal activity and minimum EC were achieved with a ***d*** of 3 mm, which we selected as the optimal needle–plate distance for the following experiments.

**Figure 3:**
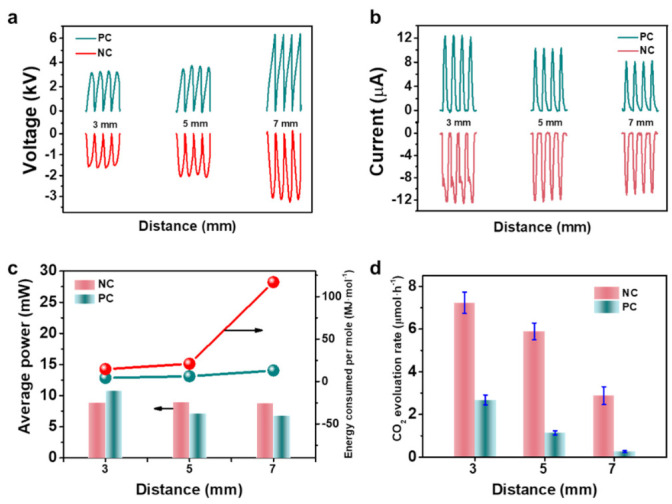
Activity dependence on the discharge polarities. (**a**) Voltage curves of different corona polarities; NC = negative corona. PC = positive corona. (**b**) Current curves of CO oxidation at different corona polarities. (**c**) Average power and energy consumption per mol CO at different corona polarities. (**d**) Activity of CO oxidation at different corona polarities. Reaction conditions: Pt as the electrode plate, CO/O_2_/He (1:20:79), 1 h discharge time, room temperature, and atmospheric pressure.

Then, we investigated the effect of the corona polarity on the CO oxidation activity, as shown in Figure 3. When ***d*** was 3 mm, the absolute value of the discharge voltage of the negative corona (1.6 kV) was lower than that of the positive corona (3.2 kV), and the absolute value of the discharge current of the negative corona (12.5 μA) was greater than that of the positive corona (12.3 μA). The average power of the negative corona (10.8 mW) was lower than that of the positive corona (8.9 mW). The CO oxidation activity of the negative corona (7.2 μmol h^−1^) was 2.7 times that of the positive corona (2.8 μmol h^−1^). Therefore, the calculated EC of the negative corona (4.2 MJ mol^−1^) was much lower than that of the positive corona (14.6 MJ mol^−1^). When ***d*** was 5 or 7 mm, the absolute value of discharge voltage and current and the activity trend were similar to those obtained with a ***d*** of 3 mm. The calculated average power of the negative corona was approximately equal for a ***d*** of 3–7 mm, whereas that under the condition of positive corona decreased significantly with increasing the distance. Meanwhile, the trend of the EC at different polarities proved to be irrespective of the ***d*** value. The above results imply that the negative corona afforded better activity and lower EC than the positive corona. Furthermore, we conducted cyclic experiments to evaluate the stability of the triboelectric plasma-triggered oxidation of CO, finding that the system maintained its activity for five runs (Figure S6).

### 3.3. Investigation of Dioxygen Activation by Triboelectric Plasma and Theoretical Calculations

**Figure 4:**
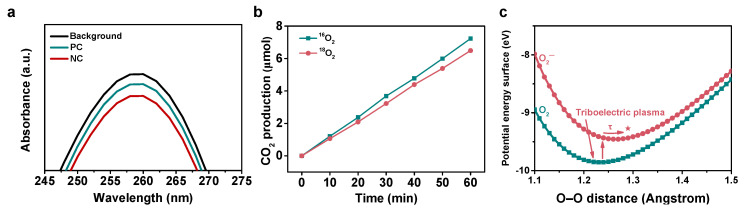
Activation of ^3^O_2_. (**a**) Reactive oxygen generated from ^3^O_2_ measured by colorimetry using a nitro blue tetrazolium solution; a.u. = arbitrary units. (**b**) Reaction rate for the triboelectric plasma-triggered CO oxidation measured at room temperature for ^16^O_2_ and ^18^O_2_ reactants. Reaction conditions: negative corona, 3 mm needle−plate distance, 400 rpm rotation speed, Pt as the electrode plate, CO/O_2_/He (1:20:79), 1 h discharge time, room temperature, and atmospheric pressure. (**c**) Density functional theory-calculated ^3^O_2_ and ^2^O_2_^–^ potential energy surface. Excitation of triboelectric plasma allows the high-energetic electrons to be transferred to ^3^O_2_, generating ^2^O_2_^–^. The ^3^O_2_ molecule is negatively charged, and the O–O nuclear motion relaxes to an equilibrium state along the ^2^O_2_^–^ potential energy surface. ★ = equilibrium state. τ denotes the progression of ^2^O_2_^–^ along the ^2^O_2_^–^ potential energy surface as a function of time.

Subsequently, to gain insight into the reason for the EC difference for the CO oxidation reaction between the positive and negative corona, we performed a nitro blue tetrazolium (NBT) assay to detect the reactive species derived from ^3^O_2_ in the triboelectric plasma by colorimetry [32], as shown in Figure 4a. Upon the reaction of NBT with ^2^O_2_^−^, insoluble blue organic compounds were generated in the reaction solution, which caused a decrease in the absorbance of the solution at 260 nm. In the absence of triboelectric plasma, the absorbance of the NBT solution decreased under CO/O_2_/He atmosphere, suggesting the existence of ^2^O_2_^−^ radicals in the triboelectric plasma. Moreover, the absorption peak under the negative corona was lower than that under the positive corona, demonstrating that the former produced more ^2^O_2_^−^ radicals than the latter.

Next, we conducted kinetic isotope experiments under the same conditions using isotope-labeled and unlabeled ^3^O_2_ to study the kinetics of the ^3^O_2_ activation. Electron-driven reactions have been reported to show higher KIEs than thermally driven and light-driven reactions [46,47,48]. As shown in Figure 4b, we used the same ratio of CO mixed standard gas (CO/^16^O_2_/He, CO/^18^O_2_/He) to evaluate the rate of CO oxidation reaction under the negative corona. The reaction rate was found to be 7.2 µmol h^−1^ under CO/^16^O_2_/He atmosphere and 6.5 µmol h^−1^ under CO/^18^O_2_/He atmosphere. The KIE (^16^O_2_ rate/^18^O_2_ rate) of the negative corona was 1.11 ± 0.01, which is larger than that reported for traditional thermal catalytic CO oxidation reactions (KIE = 1.06) [46,47,48]. The larger KIE for the triboelectric plasma process is a typical feature of an electron-driven process and confirms that ^3^O_2_ activation controls the entire reaction.

The larger KIE indicates that high-energy electrons promote the overall reaction via an electron-induced activation of ^3^O_2_. Combined with the presence of ^3^O_2_-derived reactive species as revealed by the colorimetry analysis, in the context of the reactions herein reported, we speculated that an energetic electron was directly transferred to the π* antibonding orbital (i.e., LUMO energy level) of molecular ^3^O_2_, resulting in the production of ^2^O_2_^−^, which effectively promoted the spin change and activation of ^3^O_2_. To further study the electron transfer process from the triboelectric plasma to ^3^O_2_ molecules, we determined the potential energy surfaces of ^3^O_2_ and ^2^O_2_^−^ (Figure 4c) by performing DFT and a linear expansion delta self-consistent field extension of DFT (ΔSCF-DFT), respectively. Figure 4c shows that the minimum energy required for populating the antibonding orbital of the ^3^O_2_ molecule was 0.5 eV, which corresponds to the vertical transition energy between the potential energy surface of ^2^O_2_^−^ and the ground state ^3^O_2_. Upon transferring an electron to the antibonding orbital of the ^2^O_2_ molecule, the O–O bond (the O–O bond length in O_2_ is 1.23 Å) underwent a stretching process: namely, the lowest potential energy configuration for the negatively charged ^2^O_2_^−^ ions exhibited a longer O–O bond of 1.32 Å [49].

### 3.4. Theoretical Simulation of Triboelectric Plasma

**Figure 5:**
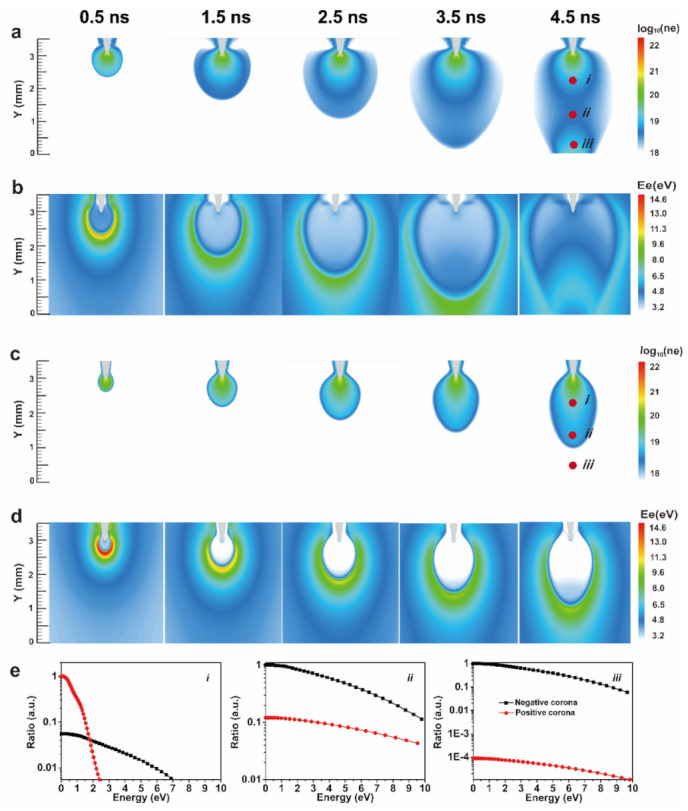
Triboelectric plasma simulation. (**a**) Time evolution of the electron density distribution in the negative corona. (**b**) Time evolution of the electron energy distribution in the negative corona. (**c**) Time evolution of the electron density distribution in the positive corona. (**d**) Time evolution of the electron energy distribution in the positive corona. (**e**) Electron energy distribution function of three representative points (i, ii, iii) both in the negative and the positive corona.

Figure 5a,b show the simulated time evolution of the electron density and the electron average energy under the negative corona with a ***d*** of 3 mm. As the time increased from 0.5 to 4.5 ns, electrons migrated from the tip of the W needle to the plate electrode, generating a plasma channel of a cylinder with a radius of 1.5 mm. The electron density was evenly distributed in the whole plasma channel, and the electron densities near the tip (point i), the middle (point ii), and the plate electrode (point iii) were 1.4 × 10^19^, 1.97 × 10^19^, and 4.9 × 10^19^ m^−3^, respectively. The average energy of electrons was evenly distributed in the whole plasma. The average energy of electrons near the tip (point i), the middle (point ii), and the plate electrode (point iii) were 3.6, 5.4, and 4.4 eV, respectively. The lower energy of electrons near the tip was mainly caused by the electric field shielding effect of the plasma.

Figure 5c,d show the simulated time evolution of the electron density and the electron average energy for the positive corona. Upon increasing the time, electrons migrated from the tip of the W needle to the plate electrode, forming a core with a radius of 1.0 mm. The electron density near the needle tip (point i) and near the plate electrode (point iii) was 1.2 × 10^18^ and 2.4 × 10^15^ m^−3^, respectively. The average energy of electrons was relatively high (about 9.5 eV) at the plasma head (point ii) and relatively low (about 0.75 eV) at the tail (point i), which was caused by the electric field shielding effect generated by the plasma.

The normalized electron energy distribution function of the three representative points (i, ii, and iii) is shown in Figure 5e. The distance between the needle tip and the three representative points was 0.6, 1.8, and 2.6 mm, respectively. The average energy of electrons at point i was lower than that at points ii and iii. For example, in point i, the energy of 100% electrons at the positive corona and 86% electrons at the negative corona was less than 5 eV. At points ii and iii, the energy of 61% electrons at the positive corona and 74% electrons at the negative corona was less than 5 eV, respectively. The energy of 73% electrons at the positive corona and 77% electrons at the negative corona was less than 5 eV. At point iii, the electron density of the positive corona was about four orders of magnitude smaller than that of the negative corona.

### 3.5. Mechanism of the Triboelectric Plasma-Triggered CO Oxidation

**Figure 6:**
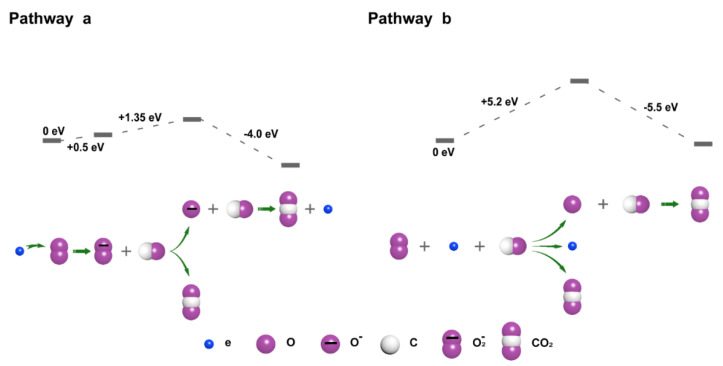
Reaction pathways for triboelectric plasma-triggered CO oxidation.

Figure 6 depicts two plausible mechanisms for the CO oxidation triggered by triboelectric plasma: pathways a and b. In pathway a, the formation of ^2^O_2_^−^ ions by changing the spin state of ^3^O_2_ allows overcoming the spin-forbidden nature of the reaction between ^3^O_2_ and CO, resulting in a low-energy barrier. This pathway includes three steps: (1) ^3^O_2_ captures low-energy electrons to form ^2^O_2_^−^ ions with a potential barrier of 0.5 eV; (2) ^2^O_2_^−^ ions react with CO to form CO and O^−^ ion, and the energy barrier is 1.35 eV [50]; (3) O^−^ reacts with CO to produce CO_2_ and e^−^ with an energy barrier of −4 eV. Pathway b comprises the direct reaction between ^3^O_2_ and CO under the action of high-energy electrons, which proceeds through a high-energy triplet transition state. This process does not overcome the spin prohibition of the reaction between ^3^O_2_ and CO; therefore, the energy barrier is high. This pathway includes two reaction processes: (1) under the action of high-energy electrons, ^3^O_2_ reacts directly with CO to produce CO_2_ and O atom, and the energy barrier is 5.2 eV; (2) The dioxygen atom reacts with CO to produce CO_2_ with an energy barrier of −5.5 eV. The total energy barrier calculated for pathway a and pathway b was 1.85 and 5.2 eV, respectively. Therefore, pathway a is thermodynamically more favored than pathway b.

**Figure 7:**
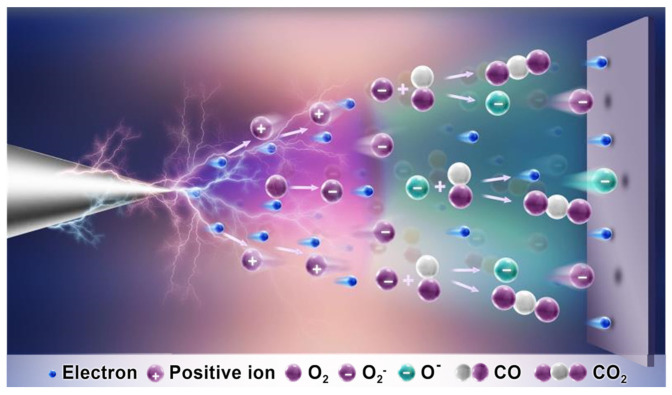
Schematic diagram of triboelectric plasma-triggered CO oxidation reaction at negative corona.

In the process of the negative corona, the average energy and average density of electrons were evenly distributed in the whole plasma channel. Except for the plasma head (near the plate electrode), the average energy of other electrons was less than 5 eV, and the energy was not sufficient for the process to proceed through pathway b, as shown in Figure 7. Therefore, in the whole plasma at the negative corona, pathway a occurs in almost all regions, whereas the process only proceeds through pathway b in the plasma head (plate electrode position). In the positive corona process, two obvious regions were formed in the plasma channel: a low-energy electron region near the needle tip and a high-energy electron region at the front edge of the plasma. The energy of electrons near the needle tip was very low; although it could produce highly active ^2^O_2_^−^ ions, it was not sufficient to promote ^2^O_2_^−^ decomposition. In the front of the plasma, 49% of the electrons have an energy greater than 5 eV, which is sufficient to overcome the energy barrier of pathway b. Therefore, in the positive corona, the oxidation of CO mainly occurred through pathways a and b. Finally, the proportion of pathway a in the negative corona was greater than that in the positive corona; therefore, the energy efficiency and activity of the negative corona were higher than those of the positive corona.

In the CO oxidation reaction, the activation of ^3^O_2_ has always been considered as the key step [51]. At room temperature, since the ^3^O_2_ molecule is in the triplet state, the oxidation needs to proceed through a high-energy triplet transition state to satisfy the spin selection rules. Therefore, although the ^3^O_2_ molecule has a strong oxidizing ability, the direct oxidation of CO is unfavorable in terms of reaction kinetics. This is the so-called spin conservation process. In thermally induced catalytic systems, the electrons of the *d* orbital of a transition metal are transferred to the antibonding orbital of ^3^O_2_ (Table S1). Meanwhile, in photocatalytic systems, after the photogenerated carriers are separated on the photocatalyst, the electrons trapped on the reduction cocatalyst migrate to the antibonding orbital of the ^3^O_2_ molecule. The ^3^O_2_ single electron transfer to form ^2^O_2_^−^ at room temperature has been reported to require metal or metal oxide catalysts. In our system, the triboelectric corona plasma contains a large number of high-energy electrons that can be directly injected into the antibonding orbital of ^3^O_2_ to produce highly active ^2^O_2_^−^, enabling the CO oxidation reaction. This system can directly realize the single electron transfer to form a ^2^O_2_^−^ free radical in the absence of a metal catalyst, which offers the advantages of low cost and no noble metal pollution [52]. The reaction device is simple and easy to operate, and the driving force of the reaction mainly comes from renewable and green mechanical energy.

## 4. Conclusions

We have developed a method that used the triboelectric corona plasma generated by a TENG under mechanical stimuli to change the spin state of O_2_ molecules and trigger the CO oxidation reaction at room temperature. The rotation speed of the TENG, the needle–plate distance, and the corona polarity have an important influence on the CO oxidation reaction. Under a rotation speed of 400 rpm, a needle–plate distance of 3 mm, and negative corona, the optimal activity was 7.2 µmol h^−1^ and the lowest energy consumption per mole CO was 4.2 MJ. The high-energy electrons in the triboelectric corona plasma were directly injected into the antibonding orbitals of O_2_ to form highly reactive O_2_^−^ radicals. The energy barrier of the reaction of the excited O_2_^−^ ions with CO is 3.4 eV lower than that of the O_2_ reaction, effectively promoting the rate-limiting step of O–O bond dissociation in the CO oxidation reaction. This work provides a novel strategy that circumvents the spin-forbidden nature of the activation of small molecules through mechanical energy.

## Data Availability

The data are available upon reasonable request from the corresponding author.

## Supplementary Materials

The following are available online at https://www.mdpi.com/article/10.3390/nano11123408/s1, Figure S1: Diagram of triboelectric plasma oxidation of CO, Figure S2: Electric curves, Figure S3: Isotope experiment, Figure S4: The effect of different rotational speeds of TENG, Figure S5: The effect of different needle–plate distances, Figure S6: Cycle experiment, Table S1: Comparison of different methods of oxidation of CO.
