# Supplementary material for: The Regulation of O2 Spin State and Direct Oxidation of CO at Room Temperature Using Triboelectric Plasma by Harvesting Mechanical Energy
Source: Nanomaterials (Basel). 2021 Dec 16;11(12):3408. doi: 10.3390/nano11123408 (PMC8703925; doi:10.3390/nano11123408)
Supplement: Supplementary file 1 [file nanomaterials-11-03408-s001.zip › nanomaterials-1489564-supplementary.pdf]

nanomaterials

Supplementary Material

The regulation of O_2 spin state and direct oxidation of CO at room temperature using triboelectric plasma by harvesting mechanical energy

Xue Shi [1,†], Sumin Li [1,†], Bao Zhang [1,†], Jiao Wang [1], Xiaochen Xiang [1], Yifei Zhu [2], Ke Zhao [1], Wanyu Shang [1], Guangqin Gu [1], Junmeng Guo [1], Peng Cui [1], Gang Cheng [1,*] and Zuliang Du [1]

[1] Key Lab for Special Functional Materials, Ministry of Education, National & Local Joint Engineering Research Center for High-efficiency Display and Lighting Technology, School of Materials Science and Engineering, Collaborative Innovation Center of Nano Functional Materials and Applications, Henan University, Kaifeng 475004, People's Republic of China; shixuehenandaxue@126.com (X.S.); amernsuli1981@163.com (S.L.); zhangbao@henu.edu.cn (B.Z.); 13203449093@163.com (J.W.); xiang17737488621@163.com (X.X.); kezhao_5@163.com (K.Z.); shangwanyu0525@163.com (W.S.); guguangqin@vip.henu.edu.cn (G.G.); junmengguo@163.com (J.G.); cuipeng@henu.edu.cn (P.C.); zld@henu.edu.cn (Z.D.)

[2] Institute of Aero-engine, School of Mechanical Engineering, Xi'an Jiaotong University, Xi'an 710049, People's Republic of China; yifei.zhu.plasma@gmail.com

* Correspondence: chenggang@henu.edu.cn

† These authors contributed equally to this work.

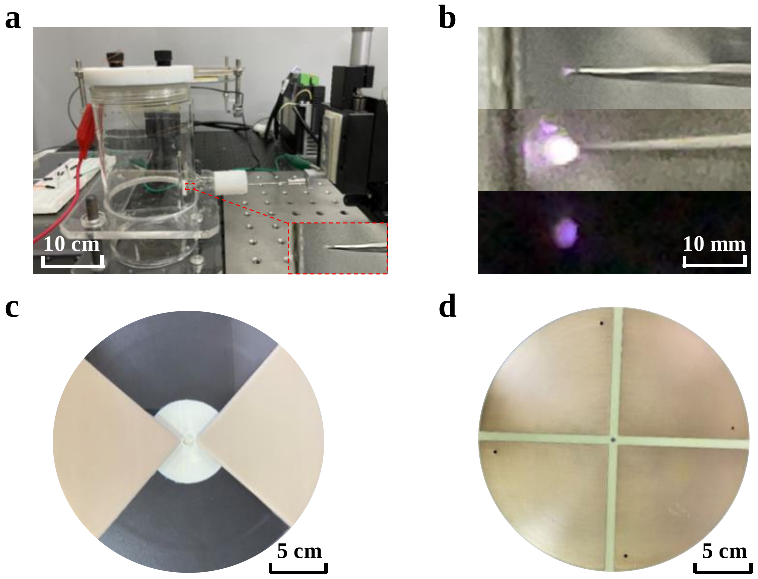

Figure S1. (**a**), Diagram of triboelectric plasma oxidation of CO at negative corona. (**b**). Triboelectric plasma at negative corona. (**c**), The rotating triboelectric layer of TENG. (**d**), The fixed metal triboelectric layer of TENG.

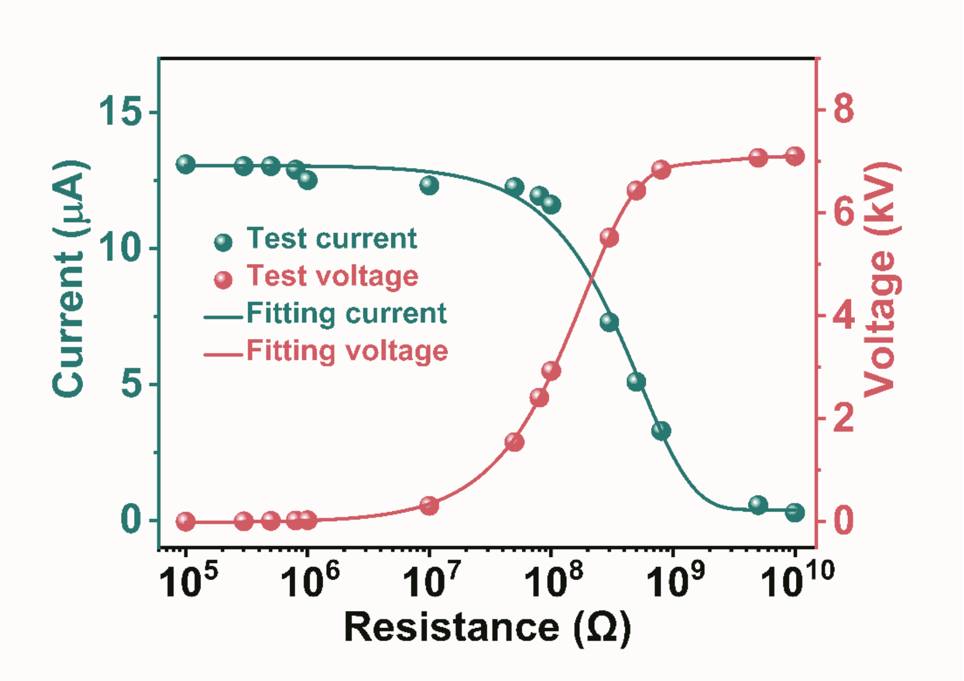

Figure S2. Voltage–impedance and current–impedance characteristic curves of the triboelectric nanogenerator.

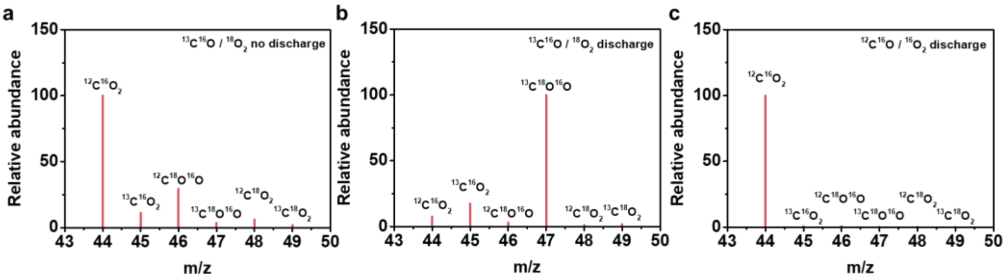

Figure S3. (**a**), Isotope exchange experiment using $^{13}C^{16}O$ and $^{18}O_2$ as substrates in the absence of triboelectric plasma. (**b**), Triboelectric plasma-induced CO oxidation using $^{13}C^{16}O$ and $^{18}O_2$ as substrates. (**c**), Triboelectric plasma-induced CO oxidation using $^{12}C^{16}O$ and $^{16}O_2$ as substrates. Reaction conditions: negative corona, 3 mm needle-plate distance, 400 rpm rotational speed, Pt as the electrode plate, $CO/O_2/He$ (1:20:79), 1 h discharge time, room temperature, and atmospheric pressure.

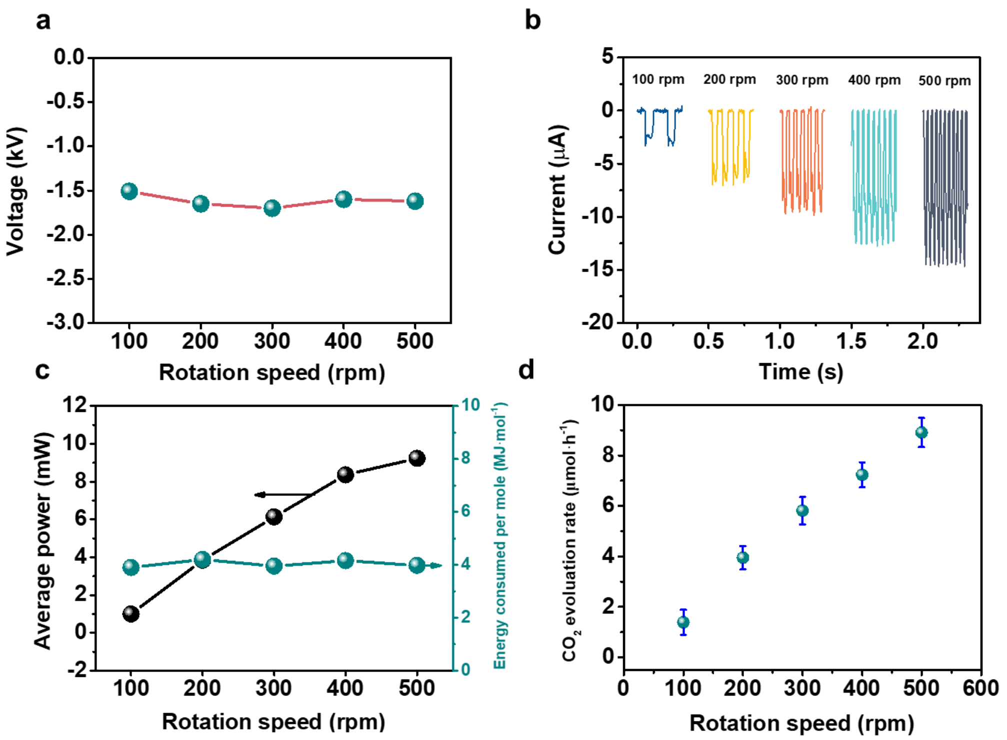

Figure S4. Triboelectric plasma-triggered CO oxidation at different rotational speeds of the triboelectric nanogenerator. (**a**), Voltage curve of triboelectric plasma at different rotational speeds. (**b**), Current curve of triboelectric plasma at different rotational speeds. (**c**), Average power and energy consumed per mole CO of triboelectric plasma at different rotational speeds. (**d**), Activity of CO oxidation at different rotational speeds. Reaction conditions: negative corona, 3 mm needle-plate distance, Pt as the electrode plate, CO/O_2/He (1:20:79), 1 h discharge time, room temperature, and atmospheric pressure.

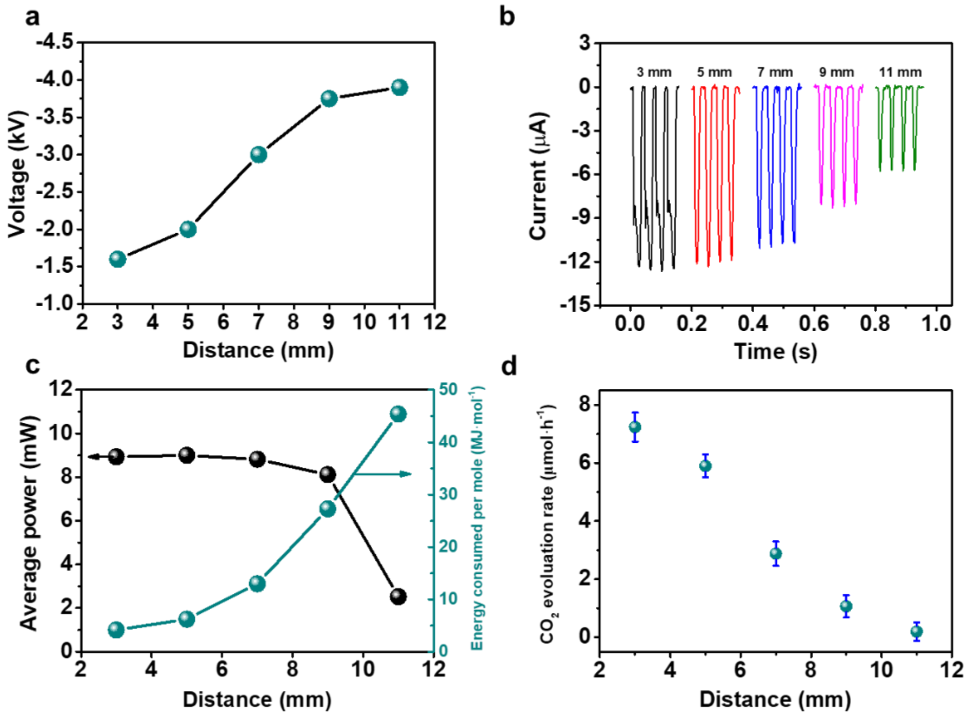

Figure S5. Triboelectric plasma-triggered CO oxidation at different needle-plate distances. (**a**), Voltage curve of triboelectric plasma at different needle-plate distances. (**b**), Current curve of triboelectric plasma at different needle-plate distances. (**c**), Average power curve and energy consumed per

mol CO of triboelectric plasma at different needle-plate distances. (**d**), Activity of CO oxidation at different needle-plate distances. Reaction conditions: negative corona, 400 rpm rotation speed, Pt as the electrode plate, $CO/O_2/He$ (1:20:79), 1 h discharge time, room temperature, and atmospheric pressure.

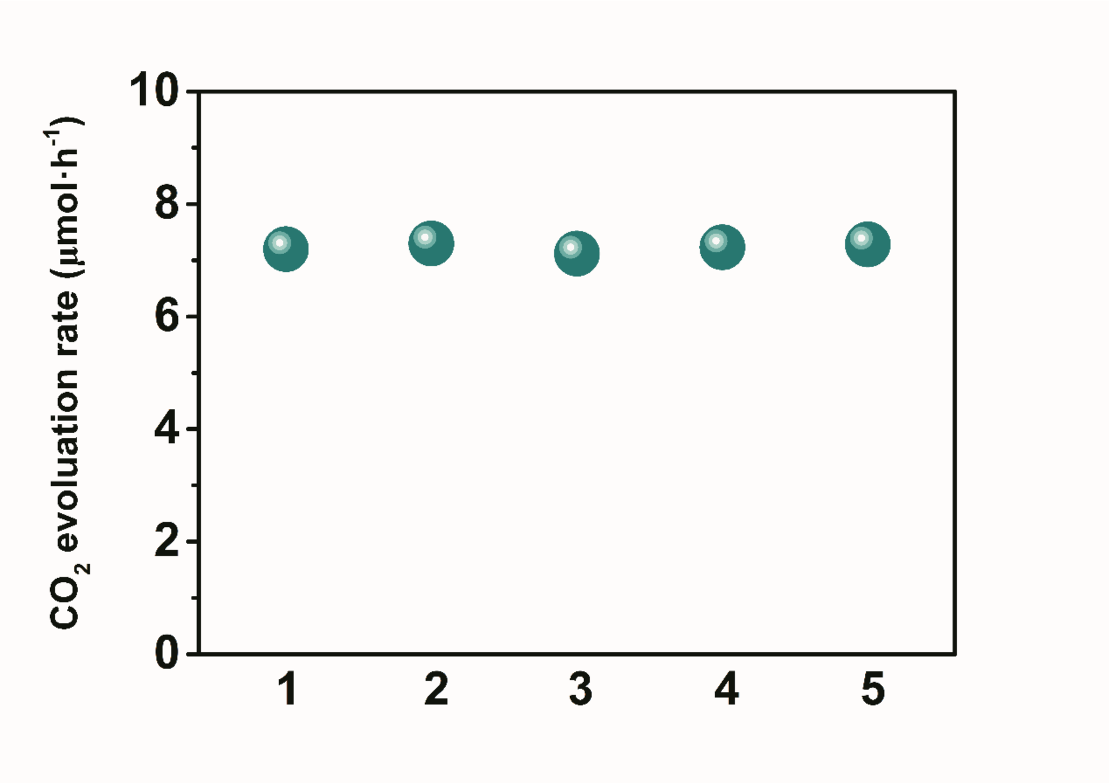

Figure S6. Cycle experiment of triboelectric plasma-triggered CO oxidation. Reaction conditions: negative corona, 3 mm needle-plate distance, 400 rpm rotation speed, Pt as the electrode plate, $CO/O_2/He$ (1:20:79), 1 h discharge time, room temperature, and atmospheric pressure.

Table S1. Comparison of different methods of oxidation of CO.

Methods of oxidation of CO	Reaction temperature	Catalyst
Thermal catalysis	100 °C	Metal/metal oxides
Photocatalysis	25 °C	Metal/metal oxides
Triboelelctric plasma	25 °C	-
